# Population-level predictors of sexually transmitted infection rate changes in Missouri: an ecological study

**DOI:** 10.1186/s13690-022-01019-6

**Published:** 2023-01-23

**Authors:** Ella Valleroy, Aaron Reed, Joseph S. Lightner

**Affiliations:** 1 University of Missouri-Kansas City, Kansas City, MO USA; 2 University of Kansas – Medical Center, Kansas City, KS USA

**Keywords:** Sexually transmitted infections, Population health, HIV

## Abstract

**Background:**

Sexually transmitted infection rates continue to increase across the US, further developing health disparities and economic burdens of disease, especially as migration occurs. In this study, we aim to assess the relationship between STI rates and population-level variables from 2008 to 2017 at the county level in Missouri.

**Methods:**

Two data sources were used: STI rates of chlamydia, gonorrhea, syphilis, HIV reported to Missouri DHSS and ACS 1-year county population estimates. Linear regression models and ANOVA tests were conducted in SPSS for each STI from year-to-year and 2008–2017. Covariates included in the analyzes were county-level income, employment rate, race, ethnicity, age, and percent poverty. Further, Akaike Information Criterion tests were performed to indicate the best predictor models and averaged standardized beta values.

**Results:**

Significant relationships among STI rates and population growth were identified. Chlamydia, syphilis, and HIV were positively associated with population growth from 2008 to 2017 (β = 0.15; β = 0.01; β = 0.05, respectively). Gonorrhea was negatively associated with population growth (β = − 0.02) but positively associated with unemployment rates (β = 0.01) highlighting the need to address population growth, as well as other variables in a population.

**Conclusions:**

There seems to be a positive relationship among population change and rates of STIs. As populations change, rates of STIs change. Moving forward, quantitative work should be conducted in various states and the nation to understand this relationship in different contexts. Future studies should be qualitative word focused on county health departments and community health improvement plans. Lastly, public policy should be implemented to buffer the impact of migration on health outcomes.

## Background

Sexually transmitted infections (STI) continue to pose a complex, significant, and constantly evolving public health concern in the United States (US) [1]. In the Midwest from 2016 to 2017, the chlamydia rate increased 5.6%, the gonorrhea rate 19.5%, and the syphilis rate 8.8% [1–3]. This increase is attributed to more widespread screening, reporting, and cases altogether [1]. Other factors include more sensitive and accurate diagnostic tests, like nucleic acid amplification tests (NAATs) [1]. A small percentage increase indicates a significant amount of new cases and subsequent healthcare costs as more are being screened and treated [1]. Human Immunodeficiency Virus (HIV) incidence has declined in recent years due to extensive preventative campaigns but has now plateaued as specific high-risk groups such as transgender persons, men who have sex with men, African American, and Latinos, are not being adequately reached, especially in the South [4].

There are serious implications if infected individuals go unscreened and untreated after contracting STIs. The reasons for not seeking diagnosis and treatment can be complex. Marginalized ethnicities, individuals that may experience racism, homophobia, and xenophobic interactions, and impoverished populations, have the lowest access to healthcare and screening tests, placing them at high risk of acquiring STIs [5]. Drug use also increases risk of STI contraction, along with unplanned pregnancies [6]. More specifically, methamphetamine use, a known problem within Missouri, increases libido and risky sex among its users [6]. Finally, group sex or sex with multiple partners can increase risk of transmission [7].

Some STIs can manifest asymptomatically more so in women and cause them to go unscreened and untreated for longer than men who typically develop symptoms faster and more noticeably [8]. For this reason, women often suffer more severe, long-term complications from STIs, some including chronic pelvic pain, ectopic pregnancies, and infertility [8].

Between screening, treatment, and long-term management of STIs, the estimated financial burden in the United States is around $15.6 billion [9]. Chlamydia and gonorrhea account for $516.7 million and $162.2 million respectively, while syphilis accounts for $39.3 million, and HIV for $12.6 billion. HIV accounts for 81% of the total annual cost of STIs in the US. The overall increase in STI cases has not been limited to a single social group, age group, gender, or socioeconomic class [7]. These costs may change as STIs shift, such as the emergence of multi-drug resistant gonorrhea [10]. Although data over the cost of STIs within Missouri is unknown, in 2016, the CDC provided $6.8 million in funding specific for HIV/Acquired Immunodeficiency Syndrome (AIDS) prevention and treatment and an additional $2.2 million in funding specific for other STIs. These values may be subject to change as multi-drug resistant gonorrhea becomes more prevalent [10, 11]. Antibiotic resistant gonorrhea has the potential to become an incurable, chronic disease resulting in disability and death in a previously easily curable infection, highlighting the need to research and intervene in STIs at a population-level now [12].

It is known that increased levels of unemployment can cause lowered income for individuals and families [13]. Increased poverty levels have been linked to increased practice of risky sexual behaviors, like using a condom inconsistently or never, not using oral contraceptives, or other forms of birth control, while also having more sexual partners and ‘one-night stands’ [13]. These behaviors increase risk of both encountering and contracting an STI [13]. Here, it can be seen that population-level variables, or macro effects, can effect STI rates and spread as populations continue to change and encounter different situations.

Social epidemiology, a research methodology and theoretical framework that focuses on social determinants, practices, and health outcomes, provides an appropriate framework to address the factors that impact STI rates. Macro effects on disease and STI transmission are often ignored and instead the focus is put on individual risk factors [7]. Social epidemiology defines three levels to the spread of STIs throughout society: 1) individual components, 2) social components, and 3) structural components [14]. Individual components involve biological susceptibility and risky behaviors. Social components involve networks, communities, and how disease diffuses across populations [14]. The structural components are grouped into 1) cultural context, 2) social networks, 3) neighborhood effects, and 4) social capital. Social epidemiology can be used as a lens to understand the impact of population change and STI rates within a state. Social epidemiology lacks the insight of an individualized point of view on STIs, but the majority of data focuses on individual trends [7]. It instead provides insight into large-scale trends affecting many with STIs, where data may be less extensive but equally compelling due to the vast amount of individuals it can then reach [7].

Given the social complexity and financial cost of STIs, it becomes critical to monitor changing rates of these infections not only throughout static populations but also as moving populations change proportions of age, gender, ethnicity, and sexuality. With these changing dynamics, estimated risks of STI transmission fluctuate as different groups have different risk factors surrounding STIs [7]. As populations grow and move, the incidence of STIs is expected to grow, showing the importance of population-level predictors alongside individual interventions. Moving, changing populations face problems of isolation and loneliness after relocating [12]. It has been observed that moving populations typically experience a higher risk of contracting STIs [15]. Characteristics of a moving population include the social disruption that accompanies geographic relocation and a lack of access to health resources [15]. STIs have remained persistent within society, in part, due to failure to contact trace all sexual partners of STI patients [9]. This is possibly due to geographic or networking barriers, consistent with a moving population. Young people and those seeking secondary education most often have to relocate, particularly from rural to urban areas [16]. In the past, the urban population of the world has increased much more quickly than those of the rural population [12]. This is expected to remain the trend into the future as the United Nations expects the world population to increase to 9.1 billion by 2100 with urban population continuing to increase in population size, and rural populations moving more towards urban areas [12]. Urban populations were considered the only “high-risk” areas for STI spread in the past, but new pockets of widespread infection in less densely populated areas have begun to form [8]. In this study, we analyze the relationship among common STIs and population change at the county level in the state of Missouri from 2008 to 2017.

## Methods

This study is an ecological study with the entire population of Missouri included in the analysis. Data was excluded if it did not address STI rates or population-level variables for the state of Missouri. A sample size calculation was not needed as data was reported and analyzed regarding the entire state of Missouri. To find the complete population count for Missouri, the Federal State Cooperative Program for Population Estimates, National Center for Health Statistics, and US Census Bureau were used. In order to understand the demographics of the Missouri population, data was taken from the Missouri Information for Community Assessment (MICA) by the Missouri Department of Health and Senior Services (DHSS) regarding county-level data on individuals aged between 15 and 44, sex, race, and ethnicity for each year between 2008 and 2017 [17]. Data on county-level households living below the poverty level was recorded from the Small Area Income and Poverty Estimates (SAIPE) Program through the Census Bureau [18].

Chlamydia, gonorrhea, and syphilis are all diseases that are required to be reported to the county health department with each new case. Each case that is reported to a county health department is required to be reported to the state health department [19]. The state health department then reports new cases of chlamydia, gonorrhea, and syphilis for each month and year. Incidence of the three STIs: chlamydia, gonorrhea, and syphilis were then compiled from the Missouri DHSS for each year between 2008 and 2017 [19]. Incidence and prevalence of HIV was obtained through Geographic Information System (GIS) programming by the Centers for Disease Control and Prevention from the years 2008–2016 [20]. At the time of this analysis, 2017 data were not available to the public. All data were exported into Excel and SPSS (version 2009, version 25) for analysis.

Analysis in SPSS and Excel evaluated the percent change in STIs from 2008 to 2017 for each county using the following formula: [(2017 County Cases / 2017 County Population) – (2008 County Cases / 2008 County population)] / (2008 County Cases / 2008 County Population). The resulting values were then exported into Table 1.

**Table 1:** Percent change in county characteristics from 2008 to 2017 -to appear between line 181 and 182

Counties	% Change White	% Change African American	% Change Latinx	% Change Avg. Yearly Income	% Change Avg. Unemployment Rate	% Change in Population Size
Adair	−3.21	89.34	21.70	10.8	−18,87	−0.09
Andrew	−1.60	80.01	56.21	13.24	−33.33	−9.02
Atchison	−1.10	0.08	41.89	10.2	−27.08	−9.02
Audrain	−1.74	7.82	34.42	8.07	−43.55	−0.52
Barry	−4.01	93.42	26.09	6.71	−40	−0.69
Barton	−2.55	104.77	66.03	12	−54.84	−5.87
Bates	−1.58	53.44	46.90	11.7	−37.5	−5
Benton	−1.40	69.16	51.88	3.1	−26.09	0.15
Bollinger	−1.10	116.78	60.17	14.3	−34.33	−0.99
Boone	−2.95	5.39	18.35	10.18	−40.91	12.77
Buchanan	−4.70	18.70	42.98	9.59	−30.77	0.85
Butler	−1.52	10.33	31.23	6.38	−27.42	0.74
Caldwell	−1.63	66.94	54.27	−2.38	−38.81	−2.77
Callaway	−0.61	−3.37	27.19	6.01	−35.19	2.68
Camden	−1.53	44.60	35.89	11.91	−23.81	4.99
Cape Girardeau	−2.59	18.66	23.53	6.29	− 32.69	5.42
Carroll	−1.02	23.39	29.87	6.57	−43.66	−6.93
Carter	−1.58	191.51	58.78	9.17	−19.44	0.77
Cass	−1.98	19.88	18.13	10.98	− 41.67	5.74
Cedar	−1.48	159.69	52.51	8.57	−36.92	−0.33
Chariton	−1.22	16.11	109.33	5.53	−43.55	−4.46
Christian	−0.97	29.63	19.57	8.82	−38	14.36
Clark	−0.85	58.21	78.59	5.32	−8.2	−6.21
Clay	−4.20	34.85	22.86	3.99	−30.77	12.89
Clinton	−0.88	12.20	34.50	9.44	−32.2	−1.09
Cole						
Cooper	−1.16	− 2.25	60.62	0.55	−36.84	0.81
Crawford	−1.16	69.26	38.14	27.88	−43.21	−2.41
Dade	−1.30	35.45	50.63	14.26	−46.03	−4.57
Dallas	−0.92	86.15	32.28	7.55	−38.16	−1.66
Daviess	−1.50	106.55	64.45	9.66	−38.89	0.37
Dekalb	−2.22	8.09	55.89	22.08	−38.1	−1.91
Dent	−1.95	49.06	102.4	5.95	−45.45	−0.1
Douglas	−1.74	140.53	115.58	9.1	−32.35	−3.62
Dunklin	−3.33	7.76	31.99	4.86	−17.72	−6.34
Franklin	−0.96	16.84	36.49	6.43	−52	2.16
Gasconade	−1.21	157.73	50.49	3.58	−50	−3.81
Gentry	−2.16	129.76	244.28	23.52	−37.78	−2.07
Greene	−2.43	21.92	32.06	9.31	−39.22	6.98
Grundy	−1.74	90.99	34.78	8.4	−35.71	−2.42
Harrison	−1.53	85.70	50.10	15.25	−28.57	−4.82
Henry	−1.91	40.65	62.03	−5.31	−38.81	−2.57
Hickory	−1.71	130.59	95.32	10.27	−53.61	−1.22
Holt	−1.20	169.76	66.01	15.24	−42	−10.41
Howard	−0.62	−2.74	32.76	7.93	−36.67	0.01
Howell	− 0.84	40.06	20.47	6.11	−17.54	0.48
Iron	−1.49	40.96	57.31	−10.68	12.28	−4.13
Jackson	−2.29	−0.82	17.18	7.94	−36.23	5.03
Jasper	−3.02	20.60	26.42	8.62	−34.62	4.41
Jefferson	−1.03	28.21	31.65	10.64	−47.83	3.42
Johnson	−3.13	21.36	50.26	2.45	−16.98	3.87
Knox	−0.78	71.03	18.74	5.3	−31.91	−3.4
Laclede	−1.27	32.93	29.31	8.35	−36.84	−0.21
Lafayette	−1.08	−0.62	37.07	7.99	−37.7	−2.37
Lawrence	−2.43	82.82	27.15	12.05	−27.08	−0.19
Lewis	−1.12	9.23	29.29	12.89	−29.17	−2.25
Lincoln	−0.95	3.78	28.27	15.1	−53.85	8.36
Linn	−1.57	35.58	68.96	5.1	−17.14	−4.64
Livingston	−2.83	49.61	78.04	4.05	−44.23	1.63
Macon	−1.02	9.94	62.32	4.11	−40	−1.68
Madison	−1.69	112.43	31.76	− 1.15	−26.23	−1.23
Maries	−2.00	179.07	51.39	17.33	−31.75	−3.53
Marion	−0.86	1.49	30.70	29.28	−35.71	0.29
Mcdonald	−4.87	260.75	9.16	4.49	−24.49	−0.5
Mercer	−2.59	103.81	249.38	8.53	−22.92	−1.87
Miller	−1.21	62.08	37.66	4.34	−34.33	1.45
Mississippi	−2.82	5.15	44.39	8.11	−31.51	−3.56
Moniteau	−1.25	8.12	21.19	16.2	−34.55	4.14
Monroe	−1.26	0.70	99.53	0.92	−38.57	−5.95
Montgomery	−1.25	8.18	49.43	14.47	−54.17	−6.8
Morgan	−1.53	53.82	45.99	15.3	−34.67	−2.89
New Madrid	−1.51	1.66	83.57	−12.98	−7.04	−6.5
Newton	−2.54	41.36	36.54	−20.74	−34.55	1.2
Nodaway	−1.57	31.69	39.21	9.89	−14.29	−2.41
Oregon	−1.55	235.12	60.15	11.61	−22.03	−1.3
Osage	−0.85	92.58	61.32	14.08	−51.61	−0.66
Ozark	−1.20	131.17	36.75	11.94	−4.92	−5.47
Pemiscot	−1.34	−0.43	41.30	−3.91	0	−9.3
Perry	−1.48	77.46	50.67	11.04	−41.3	1.31
Pettis	−3.29	7.52	33.20	7.49	−32.26	2.39
Phelps	−2.26	16.43	30.18	8.72	−37.29	1.23
Pike	−0.65	1.03	19.94	5.4	−40.35	− 0.19
Platte	−4.82	31.10	32.69	5.12	−33.33	16.3
Polk	−1.81	41.12	40.61	5.94	−34.43	3.26
Pulaski	−5.05	2.54	31.63	7.89	−24.56	7.78
Putnam	−2.04	129.92	175.79	4.29	−29.63	−4.32
Ralls	−1.25	59.85	43.54	6.76	−41.51	1.06
Randolph	−1.15	0.79	32.98	5.66	−26.23	−2.55
Ray	−1.36	26.46	47.03	7.24	−25	−3.83
Reynolds	−1.98	98.20	63.20	44.4	−46.58	−6.18
Ripley	−1.05	123.06	44.36	4.41	−17.91	−3.38
Saline	−3.71	0.18	31.57	11.01	−36.84	−1.34
Schuyler	−1.31	94.45	130.10	3.54	−25	2.85
Scotland	−0.84	129.46	62.84	9.97	−53.03	3.01
Scott	−1.53	4.42	35.01	11.54	−33.33	−1.91
Shannon	−1.19	180.70	21.55	19.79	−22.78	−2.03
Shelby	−2.17	109.02	100.74	7.19	−35.85	−6.23
St Charles	−1.81	25.83	27.94	6.42	−47.27	11.67
St Clair	−1.50	37.45	48.24	8.9	−28.17	−4.05
St Francois	−1.24	13.14	41.96	6.58	−38.03	3.77
St Louis	2.25	−9.72	21.25	5.94	−43.33	−0.16
St louis City	−2.37	11.21	29.47	4.68	−43.59	−2.93
Ste Genevieve	−1.86	31.49	65.62	4.74	−38.98	−1.89
Stoddard	−1.22	26.12	65.82	12.41	−33.33	−2.31
Stone	−1.57	161.85	42.27	15.19	−29.49	−1.31
Sullivan	−5.19	470.74	8.910	23.09	−31.03	−7.53
Taney	−3.06	86.00	33.18	7.95	−22.08	11.35
Texas	−1.46	16.49	34.97	6.65	−28.99	0.54
Vernon	−1.40	79.97	39.81	8.21	−32.14	−2.17
Warren	−1.20	17.60	19.35	12.82	−57.33	7.5
Washinton	−0.84	8.23	48.29	7.3	−47.06	−0.36
Wayne	−1.85	151.90	96.22	16.91	−25	−0.75
Webster	−0.99	27.22	30.13	6.96	−31.58	7.59
Worth						

Represents that percent change in each Missouri county regarding ethnicity, average yearly income, average unemployment rate, and population size. Positive numbers indicate an increase in the variable. Negative numbers indicate a decrease in the variable. The larger the magnitude of the number, the larger the change was within the county

Using population change as the independent variable, unadjusted multi-variate linear regression models were conducted with HIV prevalence and incidence of HIV, syphilis, gonorrhea, and chlamydia as dependent variable year-to-year and from 2008 to 2017 in SPSS (version 24).

In SPSS, further analysis used population change as an independent variable to produce unadjusted multi-variate linear regression models with HIV prevalence and incidence, syphilis, gonorrhea, and chlamydia as dependent variables. These models showed the relationship from year-to-year and from 2008 to 2017 and preliminary findings showed a positive relationship between population change and STI rate changes.

Finally, Akaike Information Criterion (AIC) tests were conducted to define a best change predictor model for STI rates [21, 22]. The Akaike information criterion is a statistical test used to find the best change predictor models in datasets when multiple possible variables are being examined. Use of the AIC as a model fit test has been used previously in research surrounding STIs propelling it to be selected for further analysis in this study in order to find the best fit predictor model for the change in STI rates over the 10-year timeframe [23]. These variable combinations are considered significant and equally qualified predictor models if the ΔAIC, or change in Akaike Information Criterion, value is below two. Of those found to be similarly qualified change predictor models, the weights, or w_i_, and R^2^ values can then be further calculated to predict the likelihood of the significant models being classified as the best change predictor model in the dataset [24]. All models predicted to be of best fit gave similar w_i_ and R^2^ values, showing that each model can be a good predictor in the future change of STIs. Note that a small alteration was made to the analysis with HIV being reported as total cases for the years of 2008 and 2017, instead of the two distinctions of incidence and prevalence to allow for a larger, more significant sample size.

## Results

Table 1 presents the percent change of study variables from 2008 to 2017, including: race/ethnicity, average household income, average unemployment rate, and population size. Most counties showed a negative percent change in white individuals and average unemployment rates and a positive percent change in African American individuals and Latinx individuals. Table 2 shows the percent change in STIs from 2008 to 2017. Most counties showed considerable percent increases in chlamydia, gonorrhea, and syphilis. The counties show mixed positive and negative percent changes regarding HIV, possibly due to the limited sample sizes and a focus on HIV prevention.

**Table 2:** Percent change in STIs from 2008 to 2017 while controlling for population change – to appear between lines 186 and 187

	% Change Chlamydia incidence	% Change Gonorrhea incidence	% Change Syphilis Incidence	% Change HIV diagnoses	% Change HIV Prevalence
Adair	50.13	57.28	0	282.42	0
Andrew	28.54	864.06	^b^NA 0–1	0	0
Atchison	449.57	^b^NA 0–1	0	0	0
Audrain	76.70	101.04	804.67	−0.95	0
Barry	97.67	156.47	0	302.27	0
Barton	123.10	0	0	0	0
Bates	100.50	1794.77	0	121.10	0
Benton	103.70	249.49	0	266.48	0
Bollinger	44.49	236.67	0	0	0
Boone	41.38	10.40	18.24	−4.05	−18.62
Buchanan	57.41	663.53	280.11	6.45	0
Butler	54.22	236.89	−66.91	88.75	0
Caldwell	14.95	259.96	0	0	0
Callaway	57.90	75.30	581.70	−45.52	0
Camden	185.73	1042.93	0	339.53	0
Cape Girardeau	18.43	−0.83	208.28	29.93	0
Carroll	25.35	544.68	0	0	0
Carter	1190.10	0	0	0	0
Cass	58.05	312.44	104.91	88.49	0
Cedar	16.39	451.84	0	−100.00	0
Chariton	33.74	4.67	0	0	0
Christian	117.77	158.83	45.74	90.43	0
Clark	113.24	0	0	0	0
Clay	36.02	144.18	172.07	60.62	22.71
Clinton	55.99	183.09	−100.00	10.91	0
Cole					
Cooper	7.82	296.78	0	136.06	−100.00
Crawford	75.20	2154.40	0	286.19	0
Dade	49.69	57.18	0	0	0
Dallas	138.97	476.25	0	0	0
Daviess	124.17	697.03	0	0	0
Dekalb	−3.40	205.84	1.95	−66.09	0
Dent	27.90	500.58	0	0	0
Douglas	250.19	366.92	0	0	0
Dunklin	32.47	144.79	967.70	−6.39	0
Franklin	85.15	401.68	−75.53	34.49	0
Gasconade	107.92	419.79	0	−26.15	0
Gentry	147.99	512.69	0	0	0
Greene	152.78	320.65	72.35	12.48	−65.25
Grundy	19.01	130.59	0	0	0
Harrison	202.07	740.54	−100.00	0	0
Henry	96.72	146.33	207.92	85.81	0
Hickory	120.88	304.94	0	0	0
Holt	−16.28	123.25	0	0	0
Howard	11.10	−33.34	0	−100.00	0
Howell	84.04	397.59	491.11	−36.84	0
Iron	47.78	108.63	0	0	0
Jackson	0.15	27.73	122.64	−0.02	−22.80
Jasper	51.56	167.66	115.51	47.69	−100.00
Jefferson	138.32	246.30	866.93	53.71	0
Johnson	36.83	152.15	0	54.60	0
Knox	3.52	−65.49	0	0	0
Laclede	23.12	483.05	0	38.57	0
Lafayette	48.84	651.15	207.29	−25.97	0
Lawrence	134.93	213.10	0.19	33.77	0
Lewis	−28.70	53.45	0	0	0
Lincoln	100.35	161.47	−53.86	134.53	0
Linn	−28.70	−100.00	0	−100.00	0
Livingston	104.35	96.78	0	0	0
Macon	1.71	−43.49	0	0	0
Madison	1.25	102.50	0	−0.38	0
Maries	3.65	0	0	0	0
Marion	35.25	3.86	0	122.32	0
Mcdonald	69.14	1055.79	0	88.37	0
Mercer	1.90	0	0	0	0
Miller	73.79	220.35	0	−31.70	0
Mississippi	−29.55	3.69	0	72.49	0
Moniteau	63.24	284.09	−3.98	71.19	0
Monroe	6.33	−57.47	0	0	0
Montgomery	177.19	758.40	0	−33.99	0
Morgan	116.80	346.24	0	−100.00	0
New Madrid	66.38	203.98	0	43.14	0
Newton	103.45	114.10	295.27	−67.29	0
Nodaway	46.20	432.82	−100.00	6.89	0
Oregon	406.58	0	0	−100.00	0
Osage	111.40	101.33	0	0	0
Ozark	196.22	^a^NA	0	−100.00	0
Pemiscot	16.99	6.39	120.52	13.39	0
Perry	73.72	12.81	0	0	0
Pettis	35.83	−39.54	0	−6.22	0
Phelps	28.81	97.57	97.57	−24/23	0
Pike	24.56	12.71	0	0.89	0
Platte	26.80	79.64	11.78	7.83	59.30
Polk	184.60	74.32	0	313.35	0
Pulaski	8.70	10.04	−53.61	41.12	0
Putnam	−37.29	0	0	0	0
Ralls	54.61	295.81	0	0	0
Randolph	3.71	−36.48	0	−23.18	0
Ray	39.37	232.73	0	−21.67	0
Reynolds	166.45	6.58	0	0	0
Ripley	63.88	131.50	0	0	0
Saline	25.82	102.71	1.35	−8.39	0
Schuyler	36.12	0	0	0	0
Scotland	−2.92	482.47	0	0	0
Scott	14.06	0.72	0	1.41	0
Shannon	155.18	0	0	−100.00	0
Shelby	35.73	−100.00	0	0	0
St Charles	57.64	173.75	85.07	61.18	44.96
St Clair	11.66	160.55	0	0	0
St Francois	143.99	413.94	317.58	−34.98	941.96
St Louis	20.13	50.47	187.70	39.95	20.97
St louis City	−3.47	19.37	128.56	4.42	−100.00
Ste Genevieve	246.54	103.84	0	0	0
Stoddard	84.65	236.32	0	62.56	0
Stone	142.05	125.17	−49.34	164.38	0
Sullivan	8.14	332.56	0	0	0
Taney	73.75	544.96	34.71	10.46	0
Texas	40.55	397.32	198.39	−0.69	0
Vernon	55.77	0	0	−11.79	0
Warren	19.83	86.05	0	104.96	0
Washington	31.59	602.55	0.36	27.71	0
Wayne	157.50	403.80	0	0	0
Webster	97.05	160.25	85.89	27.32	0
orth					
Wright	72.99	776.57	0	−26.16	0

Represents that percent change in each Missouri county regarding the STIs chlamydia, gonorrhea, syphilis, HIV diagnoses, and HIV prevalence. Positive numbers indicate an increase in the variable. Negative numbers indicate a decrease in the variable. The larger the magnitude of the number, the larger the change was within the county

^a^HIV Prevalence and Diagnoses take values from 2008 to 2016

^b^NA indicates a change from 0 to 1 case from 2008 to 2017

Table 3 shows the results of the linear regression among STIs and population change between 2008 and 2017. The association between population change and chlamydia (β = 0.60, *p* < 0.05), gonorrhea (β = 0.50, *p* < 0.05) and syphilis (β = 0.2, *p* < 0.05) showed a significant positive association. HIV diagnoses from 2015 to 2016 was positively associated with population change (β = 0.30, *p* < 0.05). HIV prevalence showed a similar positive association (β =0.03, *p* < 0.05). As populations increase or decrease in counties in Missouri, rates of STIs increase and decrease too.

**Table 3:** Associations among STIs and population change in Missouri. – To appear between lines 192 and 193

Chlamydia					
	Standardized Beta	t test value	Significance	B Confidence Interval lower limit	B Confidence Interval Upper Limit
Population Change 08–09	0.32	3.54	0.00	0.309	0.331
Population Change 09–10	0.03	0.30	0.77	0.018	0.312
Population change 10–11	0.30	3.30	0.00	0.007	0.053
Population Change 11–12	0.15	1.59	0.12	0.137	0.163
Population Change 12–13	−0.29	−3.23	0.00	−0.267	− 0.313
Population Change 13–14	0.36	4.03	0.00	0.344	0.376
Population Change 14–15	0.59	7.69	0.00	0.567	0.613
Population Change 15–16	0.32	3.52	0.00	0.144	0.496
Population Change 16–17	0.21	2.29	0.02	0.201	0.219
Population Change 08–17	0.58	7.55	0.00	0.565	0.595
Gonorrhea					
Population Change 08–09	− 0.19	−2.08	0.04	− 0.207	0.173
Population Change 09–10	0.16	1.69	0.09	0.15	0.17
Population change 10–11	−0.05	−0.50	0.62	−0.054	0.046
Population Change 11–12	0.16	1.71	0.09	−0.155	0.165
Population Change 12–13	−0.28	−3.07	0.00	−0.271	−0.28
Population Change 13–14	0.03	0.33	0.74	0.029	0.031
Population Change 14–15	0.18	1.96	0.05	0.006	0.030
Population Change 15–16	− 0.13	− 1.40	0.17	− 0.143	− 0.117
Population Change 16–17	0.57	7.31	0.00	0.541	0.599
Population Change 08–17	0.49	5.92	0.00	0.478	0.502
Syphilis					
Population Change 08–09	−0.26	−2.86	0.01	− 0.259	− 0.261
Population Change 09–10	− 0.30	−3.34	0.00	− 0.297	− 0.303
Population change 10–11	0.10	1.10	0.27	0.099	0.101
Population Change 11–12	0.08	0.89	0.37	0.079	0.081
Population Change 12–13	0.30	3.34	0.00	0.297	0.303
Population Change 13–14	0.31	3.40	0.00	0.307	0.313
Population Change 14–15	0.10	1.10	0.27	0.099	0.101
Population Change 15–16	−0.12	0.10	0.22	−0.121	−0.119
Population Change 16–17	− 0.12	−1.33	0.19	−0.121	− 0.119
Population Change 08–17	0.24	2.59	0.01	0.237	0.243
HIV Prevalence					
Population Change 08–09	0.46	5.45	0.00	0.455	0.465
Population Change 09–10	− 0.25	−2.75	0.01	−0.286	− 0.214
Population change 10–11	0.15	1.63	0.11	0.143	0.157
Population Change 11–12	0.17	1.87	0.06	0.161	0.179
Population Change 12–13	0.29	3.24	0.00	0.284	0.296
Population Change 13–14	0.12	1.26	0.21	0.118	0.123
Population Change 14–15	0.52	6.47	0.00	0.391	0.649
Population Change 15–16	0.03	2.74	0.01	0.000	0.006
HIV Diagnoses					
Population Change 08–09	−0.40	−4.54	0.00	−0.405	−0.395
Population Change 09–10	0.12	1.24	0.22	0.119	0.121
Population change 10–11	−0.10	−1.00	0.32	−0.101	0.000
Population Change 11–12	−0.03	−0.33	0.75	−0.353	0.293
Population Change 12–13	−0.13	−1.31	0.20	−0.133	−0.127
Population Change 13–14	0.07	0.78	0.44	0.069	0.071
Population Change 14–15	0.06	0.64	0.52	0.059	0.061
Population Change 15–16	0.30	3.26	0.00	0.297	0.303

Shows the associations between population change and STIs in Missouri. If the significance level value is less than 0.05, the model is considered significant and population change and STIs are significantly related

Table 4 displays the results from the Akaike information criterion test (AIC) with the significant change predictors bolded for each STI.

**Table 4:** Akaike information criterion output – to appear between lines 194 and 195

	Model	AIC	ΔAIC	Wi^a^	R^2^
Chlamydia	**Population growth + unemployment + income**	**− 135**	**0**	**0.2893**	**0.41**
	**Unemployment + income**	**− 134.92**	**0.0887**	**0.2767**	**0.40**
	**Population change + unemployment + income**	**−133.912**	**0.0887**	**0.2768**	**0.41**
	**Population growth + population size + unemployment + income**	**− 133.10**	**1.9016**	**0.1118**	**0.42**
	Income	− 131.962	3.0279		
	Population growth + income	−131.096	3.9044		
	Population size + income	− 130.773	4.2276		
	Population growth + population size + income	− 129.617	5.3828		
	Population size	− 105.627	29.3727		
	Population growth + population size	− 104.388	30.6119		
	Population growth + population size + unemployment	− 102.558	32.4423		
	Population growth	−78.0372	56.9630		
	Population growth + unemployment	−76.0378	58.9624		
	Intercept of values	−75.4091	59.5911		
	unemployment	−73.5686	61.4316		
Gonorrhea	**Population size + unemployment + income**	**− 324.99**	**0**	**0.43552**	**0.41**
	**Unemployment + incomes**	**−324.096**	**0.8944**	**0.27848**	**0.38**
	**Population growth + population size + unemployment + income**	**− 323.004**	**1.9863**	**0.16132**	**0.41**
	Population growth + unemployment + income	− 322.153	2.8378		
	Population size + income	−317.481	7.5093		
	Population growth + population size + income	− 315.911	9.0795		
	Income	−315.172	9.8185		
	Population growth + income	−313.239	11.7515		
	Population size	−303.001	21.9892		
	Population growth population size	− 301.317	23.6733		
	Population growth + population size + unemployment	−301.243	23.7476		
	Intercept of values	− 266.903	58.0875		
	Population growth	−266.008	58.9828		
	Unemployment	− 265.217	59.7735		
	Population growth + unemployment	− 264.630	60.3605		
Syphilis	**Unemployment + income**	**− 879.245**	**0**	**0.465195**	**0.43**
	**Population size + unemployment + income**	**− 877.925**	**1.3198**	**0.240561**	**0.44**
	**Population growth + unemployment + income**	**−877.397**	**1.8481**	**0.18464**	**0.42**
	Population growth + population size + unemployment + income	−875.976	3.2695		
	Income	− 871.049	8.1963		
	Population size + income	− 870.563	8.6818		
	Population growth + income	− 869.059	10.1865		
	Population growth + population size + income	− 868.672	10.5733		
	Population size	−846.402	32.8431		
	Population growth + population size	− 844.457	34.7877		
	Population growth + population size + unemployment	−843.496	35.7494		
	Intercept of values	− 812.970	66.2754		
	Population growth	−812.585	66.6605		
	Unemployment	− 811.029	68.2165		
	Population growth + unemployment	− 810.843	68.4023		
HIV	**Unemployment + income**	**− 250.519**	**0**	**0.52521**	**0.37**
	**Population size + unemployment + income**	**− 248.600**	**1.9192**	**0.20118**	**0.37**
	**Population growth + unemployment + income**	**−248.535**	**1.984**	**0.19477**	**0.37**
	Population growth + population size + unemployment + income	−246.604	3.9144		
	Income	−239.578	10.9404		
	Population size + income	− 238.168	12.3504		
	Population growth + income	−237.742	12.7764		
	Population growth + population size + income	− 236.476	14.0424		
	Population size	− 220.601	29.9180		
	Population growth + population size + unemployment	−219.483	31.0360		
	Population growth + population size	− 218.810	31.7084		
	Intercept of values	−197.583	52.9358		
	Unemployment	−196.471	54.0479		
	Population growth	−196.324	54.1943		
	Population growth + unemployment	−195.626	54.8925		

Where AIC is Akaike Information Criterion value, ΔAIC is the change in Akaike Information Criterion value, W_i_ is the Akaike weights value, and R^2^ is the correlational coefficient

Presents a test of model fitness for all county variables and their ability to predict STI rate changes. This table also presents the best fit models, where the selected variables do the best job at predicting the STI rate change, as indicated in bold print

^a^w_i_ and R^2^ were calculated for only those models considered to be of best fit

Table 5 further shows the model average beta values which can be interpreted to show the future change in STIs per unit of the variables included in the models. Population growth showed a positive value in chlamydia (β = 0.15), syphilis (β = 0.01), and HIV (β = 0.05) and a negative value in gonorrhea (β = − 0.03). Unemployment, which also appears in each STI’s change predictor model, showed a positive change for chlamydia (β = 0.02), gonorrhea (β = 0.01), syphilis (β = 0.001), and HIV (β = 0.02).

**Table 5:** Akaike information criterion model averages

	Intercept	Population growth	Unemployment	Income	Population size
Chlamydia Beta value	−0.34225	0.15206	0.01975	0.00001	0.00000
Gonorrhea Beta value	−0.20632	−0.02705	0.01224	0.00001	0.00001
Syphilis Beta Value	−0.02206	0.00664	0.00108	0.00001	0.00001
HIV Beta Value	−0.34963	0.05205	0.02104	0.00001	0.00001

The significance value is < 0.05 where all numbers represent standardized beta values between each STI and each variable of consideration

## Discussion

This study identified several relationships between common STI rates and population-level variables across Missouri at the county level. A positive relationship exists between population change and STI rates. This study also shows that population growth and size are as integral to predicting the change in STIs as other structural-level functions, like income and unemployment. As populations change, rates of STIs change.

Population growth and total HIV cases showed a positive relationship through model average standardized beta values (β = 0.05205). If we extrapolate these results to predict future cases, we would expect 52 newly-diagnosed or existing-when-moving-into-the-county cases of HIV if the population of a county grew by 1000 people. The lifetime cost of a single case of HIV is estimated as $304,500 in medical expenses [9]. An increase in 52 new cases of HIV would cost a county $15.83 million more in healthcare expenditure.

Population growth was positively associated with chlamydia such that an increase of 152 cases may occur per additional 1000 people (β = 0.15206). This adds $29,550 total in additional costs, if the cases are treated successfully after initial infection [9]. Costs increase to $3.5 million total when considering sequalae costs in asymptomatic cases. A large cost disparity exists between male and female cases, highlighting the need to intervene on those infected before chlamydia can spread in a county [9].

Population growth was positively associated with syphilis such that an increase of 7 cases may occur per additional 1000 people. This adds $4.963 in additional costs per 1000 people for treatment of syphilis (β = 0.00664) [9]. Costs increase significantly and vary in reflection to the syphilis progression. The cheapest option after syphilis contraction, is immediate treatment before possible disease progression.

Population growth was negatively associated with gonorrhea such that a decrease in 27 cases may occur per additional 1000 people (β = − 0.02705). Interestingly, unemployment was positively associated with gonorrhea such that an increase in 12 cases may occur for 1000 additional unemployed individuals (β = 0.01224). This adds $2598 in direct medical care. Sequalae costs are estimated near $3.5 million with a large cost disparity existing between men and women [9]. This cost should be investigated further with the emergence of multi-drug resistant gonorrhea [10]. This difference in conclusions highlights the need to consider multiple variables when evaluating the future of STIs. There is no single variable that can accurately predict the future change in STIs without fail. Population growth, size, unemployment, and income must all be considered when predicting the future of STI rates.

Table 1 shows the percent change in STI rates from 2008 to 2017 while controlling for population change. The change is predominantly positive, meaning that even as the population is increasing, STIs are increasing at faster rates than the population. Generally larger counties, like Jackson or St. Louis county, displayed smaller percent changes. For example, the most prevalent STI, chlamydia, had a 0.15 and 20.13% increase in Jackson and St. Louis county respectively. Smaller counties displayed significantly larger percent increases. For example, chlamydia cases increased 246.54 and 142.05% in Ste. Genevieve and Stone county, respectively. This shows that STIs are a problem for both small and large counties and are growing at disproportionately high rates in some smaller, more rural counties.

We continue to see populations moving for various reasons, commonly pursuing higher education in younger aged individuals [15]. Individuals who are moving from one place to the other may lack social support and consistent healthcare during and after the move [14]. Public health professionals should consider developing interventions within vulnerable moving populations.

Based on these data, we suggest the following action steps. First, public health researchers should assess how population-level changes may be related to other communicable diseases in Missouri. Second, a yearly analysis should be conducted by the Missouri Department of Health and Senior Services to understand how population change may be impacting communicable diseases in Missouri and recommendations for clinical interventions should be developed and implemented by delving into the qualitative side of the data. Lastly, clinicians and public health practitioners in areas with growing, changing populations need to screen more patients for STIs, especially those who have recently moved. On the national level, a similar analysis should be conducted to evaluate the effects of population growth, among other variables, on the nation’s STI rates.

The data for this study does not allow assessment of immigration or emigration or differences due to births/deaths, showing a limitation. Population change is a complex variable. Individuals are likely moving within the state. Additionally, rural counties could be losing population due to fewer births. These population-level data do not measure within state population change. In the future, this assessment should be conducting at the national level, while also accounting for and quantifying immigration and emigration within overall population change. This is a population-level study and results are generalizable only to the state of Missouri. The two most populous areas border other states: St Louis bordering Illinois and Kansas City bordering Kansas, making it a unique environment to study. Additionally, there may be other external variables that influence the relationships that were not accounted for in the methodology. This further shows the need for this analysis to be produced at the national level.

This study uses multiple data sets from the state of Missouri. By integrating many datasets, we are able to study how changing populations may impact health outcomes. This allows for a socioeconomic understanding of health outcomes. If healthcare and public health professionals can quantify how populations will increase and change with population change, precision public health can create targeted interventions. This may take place through evaluation and integration of this study or similar studies into Community Health Improvement Plans (CHIPs) which then develops to include intervention plans based off the findings.

This work follows the social epidemiology network, focused around the social capital subset and some neighborhood effects. Direct neighborhood effects from social isolation of a moving population may explain increasing rates of STIs in growing communities with social ties disrupted. Indirect neighborhood effects of increased unemployment may explain the further movement of populations in search of work, with STIs spreading alongside. More research should be done on these possible implications for social theory. Authors of this manuscript plan to conduct further analyzes using additional covariate data to test the relationships among social capital, neighbor effects, and communicable disease. More interventions and research must also be conducted on policies, Community Health Improvement Plans (CHIPs), laws, and economic factors involved in potential prevention methods for STIs. Population-wide plans should be developed and implemented to benefit the community and reduce the health and economic burden of STIs. STIs continue to pose a growing problem across the United States, Midwest region, and the state of Missouri. Prevention and response are of utmost importance as HIV continues to spread and multi-drug resistance in gonorrhea becomes more apparent in the population. Population-level predictors are imperative to understanding STI rate changes and predicting when they may occur so that intervention may be put into place. As populations change in Missouri, due to movement, unemployment rates, or other variables, STIs change with them. Health care and public health providers should respond to moving populations and individuals through screening for STIs and implementing preventative measures. Missouri law and policy makers should develop legislation to better support moving populations and those facing unemployment in order to limit the spread of STIs. Further, legislation should be drafted to prevent unemployment rates from increasing and making preventative measures, like condoms and sexual education, more widespread. This way, the health and wealth of Missourians and United States residents may be bettered.

## Conclusion

As population compositions, economic conditions, and prevention efforts change and grow, STI rates change and grow with them. Population size and growth are instrumental in predicting large-scale changes in STIs. Income, unemployment, and poverty rates must also be considered when evaluating STIs in a large population in order to predict future changes and emphasize prevention efforts as populations shift.

## Data Availability

The datasets generated or analyzed during this study are available from the corresponding author on reasonable request.

## Abbreviations

STI: Sexually Transmitted Infection
US: United States
NAATs: Nucleic acid amplification tests
HIV: Human Immunodeficiency Virus
AIDS: Acquired Immunodeficiency Syndrome
MICA: Missouri Information for Community Assessment
DHSS: Missouri Department of Health and Senior Services
SAIPE: Small Area Income and Poverty Estimates
GIS: Geographic Information System
AIC: Akaike Information Criterion
ΔAIC: Change in Akaike Information Criterion
w_i_: weights
CHIPs: Community Health Improvement Plans
